# Acute renal allograft dysfunction due to cecal volvulus: a case report

**DOI:** 10.1186/s40064-015-1229-7

**Published:** 2015-08-22

**Authors:** Sherry-Ann N. Brown, Patrick G. Dean, LaTonya J. Hickson

**Affiliations:** Division of Internal Medicine, Department of Medicine, Mayo Clinic, Rochester, MN USA; Division of Transplantation Surgery, Department of Surgery, Mayo Clinic, Rochester, MN USA; Division of Nephrology and Hypertension, Department of Medicine, Mayo Clinic, 200 First Street SW, Rochester, MN 55905 USA

**Keywords:** Acute kidney injury, Ureteral obstruction, Volvulus, Kidney transplant

## Abstract

**Purpose:**

Among kidney transplant recipients with acute kidney injury, the differential diagnosis must be broadened to include conditions such as rejection, immunocompromised host infections, anatomic pathologies, and recurrent or de novo glomerular diseases. In this case report, we describe an unusual cause of acute renal allograft injury due to external compression of the allograft ureter.

**Methods:**

Retrospective review; case report.

**Results:**

The patient developed acute kidney injury of the renal allograft due to external compression of the allograft ureter coincident with a cecal volvulus. The patient underwent lysis of adhesions, right hemicolectomy, and end ileostomy creation with resolution of acute kidney injury.

**Conclusions:**

Cecal volvulus is an uncommon cause of bowel obstruction and is often associated with adhesions following abdominal surgery. To our knowledge, cecal volvulus has not previously been reported as a direct contributor to acute kidney injury. This case highlights the need for a systematic approach to the patient with acute kidney injury and the special considerations involved in the diagnosis of renal failure in the kidney transplant population.

## Background

Acute kidney injury (AKI) occurs in approximately 1 % of all hospitalized patients and is often multifactorial in etiology (Thadhani et al. 1996). Among patients with AKI, mortality rates may range between 45 and 70 % in the hospital and intensive care unit settings (Thadhani et al. 1996). Kidney transplant recipients represent a unique population highly susceptible to AKI. In this group, the incidence of AKI is 30–50 % during the first 3–6 months following transplantation (John and Herzenberg 2010). The evaluation of AKI in recipients of kidney transplant must encompass not only the differential of AKI for native kidneys but also the effect of a chronically immunosuppressed state, toxicities from nephrotoxic drug exposure, and immunologic causes (Hickson et al. 2009; Stegall et al. 2011).

A structured approach to AKI assessment can expedite diagnosis, direct early appropriate treatment, and minimize the associated morbidity and mortality. Such an approach classifies sources of AKI into 3 distinct categories: prerenal, renal, and postrenal. Prerenal causes are generally hypotension, ischemia, or another cause of reduced effective renal perfusion. Renal causes include tubular, interstitial, or glomerular injury from glomerulonephritis, interstitial inflammation, or infiltrative processes. Lastly, postrenal causes are generally anatomic abnormalities that lead to obstruction of the urinary outflow tract. The history and physical exam should be targeted at the identification of exacerbating factors. Laboratory testing, including kidney biopsy depending on the clinical situation, and imaging studies of the kidneys are often indicated to elucidate the cause and direct therapy.

We present an unusual cause of acute renal allograft injury due to external compression of the allograft ureter coincident with a cecal volvulus which illustrates the complexity of acute kidney injury diagnosis and management in the kidney transplantation.

## Case report

A 60-year-old woman with lupus nephritis and ischemic cardiomyopathy developed emesis, abdominal discomfort, diarrhea and acute renal allograft dysfunction 6 months after combined heart-kidney transplantation. The most recent kidney transplant was her third and was performed through a midline abdominal incision. The right common iliac artery and vein were used for inflow and outflow, respectively. Maintenance immunosuppression included tacrolimus, mycophenolate mofetil, and prednisone. Fluconazole was recently re-initiated for prophylaxis against fungal infections.

Physical examination was remarkable for blood pressure 98/64 mmHg, pulse 111 bpm, minimal skin tenting, mild diffuse abdominal tenderness including over the renal allograft, and coarse resting upper extremity tremors. Laboratory testing revealed macrocytic anemia (hemoglobin 10.1 g/dL; normal: 12–15.5) and metabolic acidemia (bicarbonate 18 mmol/L; normal: 22–29). Serum creatinine was elevated at 4.2 mg/dL (normal: 0.8–1.3; baseline: 1.0). Urinalysis was notable for mild proteinuria, hematuria, pyuria, and renal epithelial cells. Serum BK virus levels by PCR were elevated (73,300 copies/mL). A 12-h tacrolimus trough level was supratherapeutic at 27.8 ng/mL (goal: 5–8 ng/mL).

**Fig. 1:**
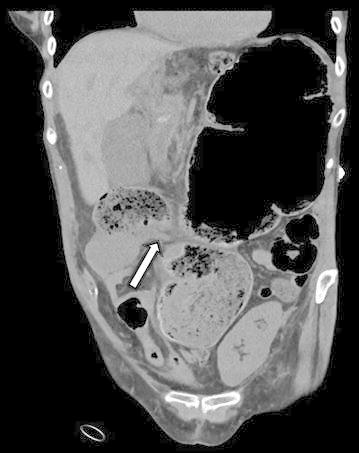
Abdominal/pelvis CT demonstrating significant dilatation of the cecum and proximal allograft ureter (*arrow*)

**Fig. 2:**
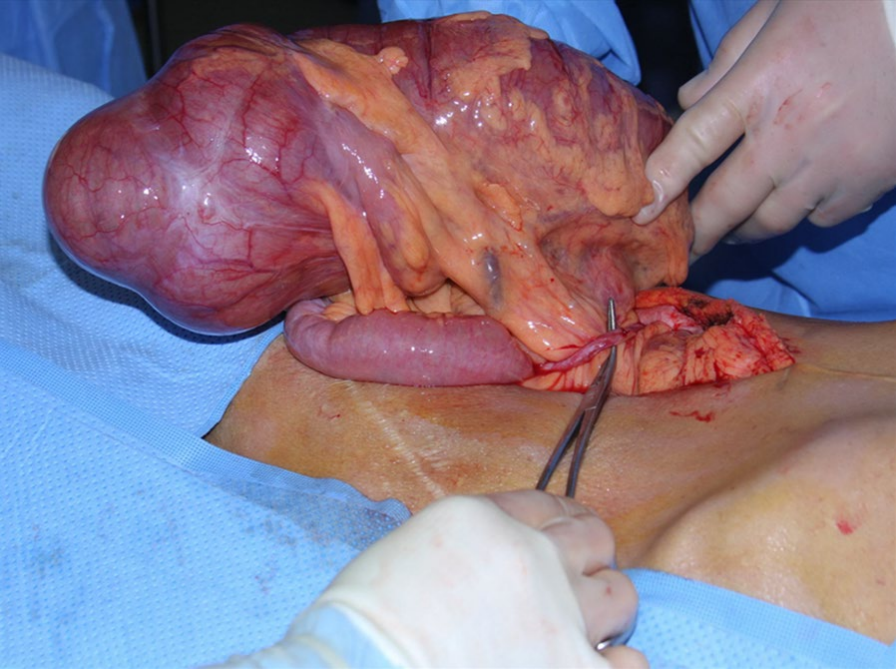
The distal small bowel and proximal colonic mesentery had herniated posterior to the intraperitoneal allograft ureter, resulting in cecal volvulus and allograft ureteral obstruction

Renal allograft ultrasound showed hydronephrosis and a CT scan of the abdomen and pelvis (Fig. 1) demonstrated significant dilatation of the cecum and proximal allograft ureter (arrow). She underwent surgical exploration. The distal small bowel and proximal colonic mesentery had herniated posterior to the intraperitoneal allograft ureter (Fig. 2), resulting in cecal volvulus and allograft ureteral obstruction. A right hemicolectomy was performed. Postoperatively, she experienced no significant complications. Tacrolimus and fluconazole were temporarily discontinued. Within a few weeks, her renal allograft function improved (serum creatinine 0.9 mg/dL) and BK virus levels were reduced in the setting of reduced immunosuppressive drug target levels.

## Discussion

This patient’s AKI was multifactorial and encompassed prerenal, renal, and postrenal causes. The prerenal contribution resulted from poor oral fluid intake, which responded to rehydration with normal saline. Renal components included acute tubular necrosis (ATN) and drug-associated injury from supratherapeutic tacrolimus levels (in part due to gastrointestinal disturbances and perhaps due to drug–drug interactions with fluconazole) leading to vasoconstriction and tubular toxicity. The ATN, a diagnosis supported by the presence of renal epithelial cells on urine microscopy, was conservatively treated with avoidance of further hypotension, ischemia, or low renal perfusion, and a reduction of calcineurin inhibitor therapy. The most surprising etiology of her AKI was postrenal, due to obstructive nephropathy from compression of the allograft ureter coincident with the cecal volvulus. Thus, several interventions were pursued to address this patient’s multifactorial allograft AKI.

To our knowledge, this is the first report of acute allograft dysfunction due to cecal volvulus. Cecal volvulus is uncommon, accounting for 1 % of bowel obstructions (Rosenblat et al. 2010), and confers high mortality (Eng and Ravindra 2009). Patients may present acutely or insidiously, based on the degree of obstruction or ischemia, leading to complaints of nonspecific abdominal discomfort, possibly associated with nausea, vomiting, or constipation (Madiba and Thomson 2002). Typically, cecal volvulus occurs in middle-aged, female patients (Rabinovici et al. 1990; Ballantyne et al. 1985). Among risk factors cited are adhesions, malrotation, colonic distension, and upward displacement of the cecum (Rakinic 2011). Cecal volvulus may occur early after retroperitoneal operations that disrupt the normal retroperitoneal attachments of the right colon (Rakinic 2011). For example, cecal volvulus though infrequent has been reported within days of laparoscopic nephrectomy and renal transplantation (Eng and Ravindra 2009), nephroureterectomy in a living kidney donor (Etheredge et al. 1977), radical nephrectomy for renal cell carcinoma (Scott et al. 2008), and palliative nephrectomy (Ali Khan et al. 1985).

Renal allograft complications often lead to allograft dysfunction or loss. These may include acute or chronic forms of rejection (cellular and/or antibody mediated), ischemic injury, calcineurin inhibitor toxicity (including thrombotic microangiopathy), pyelonephritis, infections common to immunocompromised hosts (including polyoma virus nephropathy), drug-related interstitial nephritis, recurrent or de novo glomerular diseases, among others. Adding to the complexity of care is the potential for heightened injury from nephrotoxic drug exposure. In this case, fluconazole was likely a contributing factor to the supratherapeutic tacrolimus levels due to a drug–drug interaction with tacrolimus. Drug-drug interactions should be considered at each instance of medication reconciliation, prescription, or discontinuation to avoid supratherapeutic or subtherapeutic immunosuppressive drug levels, due to varying activity of the cytochrome P450 liver enzymes. Azole antifungals classically increase calcineurin inhibitor drug levels, as is suspected in the case of our patient. Systematic assessment of known or potential causes of acute renal allograft dysfunction can uncover previously unsuspected etiologies and help prevent worsening of this condition that carries significant mortality. Finally, anatomic abnormalities may contribute to renal allograft injury. Ureteral obstruction complicates approximately 2 % of renal allograft injuries, most frequently caused by anastomotic stricture or ischemia-induced fibrosis (van Roijen et al. 2001). Other rare causes have been reported, such as inguinal herniation of the allograft ureter (Ingber et al. 2007).

## Conclusion

In conclusion, the diagnosis and management of acute kidney injury in the kidney transplant recipient requires expansion of the differential diagnosis beyond that used for non-transplant recipients. Careful physical exam, history taking, and laboratory studies are often complimented by imaging tests to help elucidate a cause. In this case, complications related to the recent transplant operation resulted in an unusual source of kidney injury.
